# Editorials

**Published:** 1910-09

**Authors:** 


### EDITORIALS

The Business Office of the *JOURNAL-RECORD* is 1 1-2 to 5 1-2 South Broad Street.
The Editorial Office is 1014-15 Century Building.
Address all Business Communications to *Journal-Record of Medicine*, 1 1-2 to 5 1-2 South Broad Street.
Make remittances payable to *THE JOURNAL-RECORD OF MEDICINE.*
On matters pertaining to the Editorial and Original Communications, address Edgar G. Ballenger, M. D., Atlanta, Ga.
Reprints of Original Articles will be furnished at cost price. Requests for the same should always be made in THE MANUSCRIPT.
We will present, postpaid, on request, to each contributor of an original article, twenty (20) marked copies of *THE JOURNAL-RECORD OF MEDICINE* containing such article.

### JUNOD'S BLOOD DERIVATIONS.

Werber * has recently called attention to the ingenious studies and devices of Victor Theodore Junod, who received his doctor's degree in Paris in 1833. His method of treatment was quite identical with our modern treatment by hyperemia as advocated by Bier, except Junod exhausted the blood from and not into the inflamed or diseased tissues and organs as does Bier, although it seems that Bier has not given Junod due credit for his work which was distinctly original in every way and anti-dated Bier's work more than a half century.

As logical and reasonable as Junod's method seems, it is strange that it should have been so completely overlooked in later years. Air-tight receivers were applied over the arms and legs, either singly or in pairs, or all at one time, and a suitable pump was used to exhaust the air from one-eighth to one-quarter (minus) atmosphere. This caused an enormous local influx of blood and fluids, and an equally great reduction of congestion in diseased areas. The imprisoned serum and blood were held in the limb or limbs for a few minutes to a few hours according to the cause of the affection being treated; the most prolonged treatments having proved the most effective.

Weber states that: "Junod claimed for this operation the lowering of the internal and external temperature of the body, causing abundant perspiration; the cure of diseases characterized by

* Junod's Blood Derivations—Read Before the Nineteenth Annual Meeting of the American Electro-Therapeutic Association, in New York, September 29, 1909.

inflammation, and that it afforded invariable relief for local congestions; also that by a timely application fever was aborted, and that it quieted even the wild delirium of mania. He taught that his derivations had a rapid sedative effect, which is very usetul in the treatment of many cerebral, thoracic and abdominal affections; also that they were attended by abundant and continuous perspiration, which plays a critical part in the treatment of certain affections. Junod's derivations usually produced sleep, or, at least, a calm, placid condition. Inflammation was subdued *turae*. but he gives no additional suggestion on this point."

This critical sweat kept up in many cases for twenty-four hours or more and was regarded a valuable process to cleanse the blood of pathologic products inimical to health.

By diminishing the mass of fluid in circulation Junod hastened the absorption of serum in other parts of the body, to overcome the action of an excited heart and disperses inflammatory congestions.

Junod states that it is certain that in most cases his derivations employed with persistence and moderation act successfully by the renewed impulse they give to the circulation in combatting sanguineous stagnation if there is either active or passive congestion, and in re-establishing the equilibrium between the contending forces of composition and decomposition if there is a defect of nutrition.

That Junod's procedure would drain the "stagnant pools" in that a kind of counter stimulation is established which quiets this constantly diminishing blood pressure induced in the larger blood vessels. The renal and intestinal secretions were increased, and in many instances, he says, as much as though diuretics and purgatives had been administered.

We have here a hint that his derivations stimulate to increased activity the physician's time-honored ally, the *vis medicatrix na-* nerve centers.

Profuse perspiration after Hæmospasia (to draw blood) denotes salutary crisis from influencing the nervous system, and the body seems not unreasonable to believe, and thus flush infected areas with a fresh supply of fluids and blood. In this respect it seems to meet the modern requirements for the cure of many affections and should find even a greater field of usefulness than does the present method of artificially induced hy peremia.

---

### THE AFTERMATH.

Considerable interest was excited a few years ago at the efforts of the now defunct *Morning World* in educating the public to the evils attending the patronage of advertising physicians. The efforts of the *World* were stopped by the demise of the paper, but the movement so begun established the interest of the Federal authorities, with the result that three notable instances of advertising specialists have been convicted and sentenced for using the United States mail with intention to defraud.

The ban of the medical profession has been laid at all times against persons following the practices common to the advertising practitioner of medicine, particularly where pretentious claims are made to the accomplishment of results which are doubtful, to say the least.

The general public has been slow to appreciate their victimiation, and it has required the interference of a paternal Government to justify the attitude of the medical profession.

The lines of least resistance are not always those which are either the most honest or those of the best policy, and sooner or later the evil-doer is liable to be overtaken. All urban communities are afflicted with too many advertising practitioners of medicine who submerge the professional instinct in the desire for gain, and it is to be hoped that the incident of Federal action in the case of the New Orleans specialists may serve as a precedent to quicken the action of the authorities in other communities.—New Orleans Medical Journal.

